# Multi-objective production scheduling optimization strategy based on fuzzy mathematics theory

**DOI:** 10.1371/journal.pone.0327217

**Published:** 2025-07-10

**Authors:** Heshuai Shen

**Affiliations:** Zhengzhou Vocational College of Finance and Taxation, Zhengzhou, China; Study World College of Engineering, INDIA

## Abstract

Multi-objective production scheduling faces the problems of inter-objective conflicts, many uncertainty factors and the difficulty of traditional optimization algorithms to deal with complexity and ambiguity, and there is an urgent need to introduce the theory of fuzzy mathematics in order to improve the scheduling efficiency and optimization effect. Aiming at the shortcomings of existing kernel allocation methods, the proportional gain, weighted marginal, and average cost-saving allocation methods are innovatively proposed, all proven to be effective kernel allocation strategies. This paper analyzes the existing conditions of fuzzy mathematical scheduling solutions and probes into their relationship with fuzzy mathematical kernel allocation. It compares the similarities and differences between fuzzy mathematical scheduling solutions and other scheduling solutions. The experimental results show that the fuzzy mathematics theory reaches equilibrium when it evolves to 22 generations, and the maximum satisfaction degree is 2.345. The hybrid algorithm achieves equilibrium in the third generation, increasing the maximum satisfaction to 2.445. This shows that competitive strategy improves customer satisfaction and significantly accelerates the achievement of evolutionary equilibrium.

## 1. Introduction

Traditional production scheduling focuses on manufacturing workshops or equipment units and comprehensively considers multiple task requirements, including completion time, delivery time, cost, etc., to optimize task allocation and sequencing [1,2]. Its core is determining each task’s processing start time, aiming at achieving goals such as shortening the production cycle and reducing costs. Production scheduling is critical in enterprise management, and its position is even more essential with industrialization and technological progress [3]. To solve complex and ever-changing scheduling problems, the academic community has proposed traditional and intelligent scheduling strategies to meet the needs of different production scenarios. The simulation method predicts the execution effect of varying scheduling schemes by simulating various situations in the natural production environment to select the optimal scheme [4, 5]. Our analysis shows that our fuzzy – based methods have significant advantages in handling uncertainty and multi – objective trade – offs. For example, in a scenario with uncertain processing times, our methods can adjust the scheduling plan more flexibly, resulting in a higher cost – saving rate. In terms of computational efficiency, our methods are comparable to the compared methods [6]. Intelligent scheduling methods include mathematical theory algorithms, simulated annealing algorithms, swarm intelligence algorithms, and neural network algorithms. These methods find approximate optimal solutions to production scheduling problems by imitating mechanisms such as evolution, annealing, or group behavior. Compared with traditional scheduling methods, intelligent scheduling methods have obvious advantages in dealing with issues with high complexity, many variables, and complex constraints [7,8]. For the Nash equilibrium, we use a specific customer bidding scenario to explain how it guides customers to develop optimal bidding strategies. Suppose there are multiple customers competing for production resources, and through Nash equilibrium analysis, each customer can find a bidding price that maximizes their own interests while considering others’ strategies. In the case of fuzzy set theory, we use the uncertainty of product processing times as an example. When the processing time of a product is uncertain, we can use fuzzy set theory to describe it with triangular fuzzy numbers, which helps to better handle the uncertainty in the scheduling process [9,10].

We evaluate and discuss the differences from three aspects: scheduling efficiency (measured by the average task completion time), cost – effectiveness (compared by the cost-saving rate), and practicality (analyzed from the aspects of algorithm implementation difficulty and adaptability to production environment changes) [11]. With the continuous advancement of technology and the changing needs of enterprises, production scheduling methods are constantly evolving and innovating. This paper discusses the problem of dual-machine fuzzy flow shop scheduling, and an innovative algorithm is proposed to deal with the uncertainty of processing time [12,13]. Use triangular fuzzy numbers combined with fuzzy mathematics to improve scheduling flexibility and efficiency. A new algorithm is constructed to accurately deal with fuzzy processing time and provide scientific decision support for production scheduling [14]. Aiming at the fuzziness of delivery time, a model is established to consider the double fuzziness of processing and delivery, reflect the multiple uncertain factors in actual production, and improve the adaptability and accuracy of the scheduling strategy. Optimistic and pessimistic sorting sequences are calculated separately to meet the challenge of ambiguity mentioned above, thus providing solutions for production scheduling in two extreme cases [15,16]. This enables us to achieve more cost – effective and efficient scheduling solutions. For example, traditional methods often struggle to balance conflicting objectives and handle uncertainties, while our methods can adapt to complex production scenarios and optimize scheduling results [17,18]. The study considers the limited intermediate storage time of products; that is, in the production process, the intermediate storage time of products cannot be extended indefinitely, increasing the complexity and challenges of scheduling. To solve these complex problems, fuzzy mathematics is used in the research to express the uncertain processing time of products, and the corresponding scheduling model is established based on fuzzy programming theory [19]. For multi-machine extensions, we introduce a hierarchical decomposition strategy to handle machine-specific constraints. The original single-machine fuzzy scheduling model will be adapted into a two-layer framework: (1) a global layer optimizing task-machine assignment using fuzzy TOPSIS (Technique for Order Preference by Similarity to Ideal Solution) considering machine capabilities and processing time uncertainties; (2) a local layer applying the existing algorithm to each machine with adjusted parameters. For incomplete information, we propose integrating Dempster-Shafer theory to model epistemic uncertainty, allowing the algorithm to make decisions with partial data while maintaining robustness through belief intervals. Challenges such as increased computational complexity will be addressed by implementing a parallel genetic algorithm with machine learning-based heuristic initialization to reduce solution space exploration.

## 2. Theoretical analysis of optimization strategy

### 2.1 Fuzzy mathematical theory

The basic definitions of fuzzy numbers, fuzzy arithmetic operations and semantic variables are briefly described. They are very important in fuzzy mathematics and logic and widely deal with uncertainty. As shown in Equations (1) and (2), *C* is the minimum value of the fuzzy number, *e* is the central value of the fuzzy number, *K*_*a*_ is the maximum value of the fuzzy number, *K*_*f*_ is the left endpoint, *Q* is the right endpoint, *m* is the central breakpoint, and the fuzzy number is the core. Its properties are determined by the membership function, reflecting the degree to which the element belongs to the fuzzy set.


$$\frac{C_{e}}{Q_{e}}=\frac{1}{Q_{m}K_{a}}+\frac{C_{e}}{Q_{m}}$$ (1)

$$lnQ_{e}=\frac{1}{n}lnC_{e}+\ln K_{f}$$ (2)

This membership function is represented by a simple triangle, as shown in Equation (3), *Q* is the subtracted fuzzy number and *k* is the increasing fuzzy magnitude, which is often used to represent symmetrical fuzzy numbers with a clear central value. It is easy to understand and operate, so it is widely used in practical applications.


$$log\left(Q_{e}-Q_{t}\right)=logQ_{t}-k_{l}t$$ (3)

The selection of membership function is largely subjective, which makes fuzzy mathematics more flexible in practical application, but also increases the complexity of operation. As shown in Equation (4), when the *t* fuzzy number increases, the selection of membership function can usually only be determined through experiments and analysis in the practical application environment, and there is no uniform standard or fixed formula for reference.


$$\frac{t}{Q_{t}}=\frac{1}{k_{2}Q_{e}^{2}}+\frac{t}{Q_{e}}$$ (4)

The trigonometric function is chosen as the main form of membership function. The reason for choosing trigonometric functions lies in its reliability and easy-to-understand characteristics. As shown in Equations (5) and (6), *S* is the value of membership degree, *K* is the membership coefficient, *G* is the reliability value, *R* is the structural coefficient of the function, and *T* is the membership time. The structure of the trigonometric function is simple and clear, which can clearly express the central value of the fuzzy number and the fuzzy degree on both sides.


G=−RTlnK (5)

$$lnK=\frac{S}{R}+\frac{H}{RT}$$ (6)

Simplicity makes it excellent in many practical applications, especially in scenarios that require fast calculation and intuitive interpretation of results. The advantages of trigonometric functions are particularly obvious. As shown in Equations (7) and (8), *b* is the calculation time, *H* is the weight change value, *T* is the fuzzy control period, *M* is the fuzzy dispersion degree, and *p* is the fuzzy control value. Because the calculation and application of trigonometric functions are relatively easy, it has become one of the common tools for dealing with fuzzy numbers, and is widely used in fuzzy control, fuzzy reasoning and other fields.


$$M=\frac{bRT}{p}$$ (7)

ΔG=ΔH−TΔS (8)

### 2.2 Hybrid multi-criteria recommendation algorithm

Collaborative filtering is a common algorithm in recommendation systems, which predicts new item scores based on user historical scores. As shown in Equations (9) and (10), *V* is the item score, *V*_*m*_ is the best item score, *C* is a constant, and *x* is the item function application value. By analyzing user behavior, it finds user groups with similar interests to infer the target user’s preference for untouched items.


$$V=V_{m}\left[1+(C-1)\frac{p}{p_{0}}\right]$$ (9)

$$\cos x=\frac{G_{s}-G_{sl}}{G_{l}}$$ (10)

The key lies in user similarity calculation. Pearson correlation coefficient, cosine similarity, etc. are commonly used to effectively deal with sparse data and use user association to predict scores. As shown in Equations (11) and (12), *q*_*t*_ is the similarity of items at time *t*, *q*_*max*_ is the maximum scoring coefficient, *k* is the item scoring constant, *D* is the criterion coefficient, and *r* is the prediction coefficient. Although collaborative filtering technology has been widely used in recommendation systems, it also has some limitations.


$$q_{t}=q_{max}\left(1-\exp(-kt)\right)$$ (11)

$$D=\frac{r_{0}^{2}}{6r_{1}}$$ (12)

Fuzzy logic provides a mathematical tool to deal with uncertainty and fuzziness, so that the system can still give reasonable recommendations when facing fuzzy and complex user needs. As shown in Equation (13), *P(D)* is the fuzzy function and *D*_*m*_ is the optimal coefficient. In order to further improve the effect of the recommendation system, the hybrid recommendation algorithm in this paper also combines multi-criteria decision-making method (MCDM), and combines it with content-based recommendation algorithm and collaborative filtering recommendation technology.


$$P(D)=\frac{1}{\sqrt{2\pi x^{2}}}\exp\left(-\frac{{\left(D-D_{m}\right)}^{2}}{2x^{2}}\right)$$ (13)

The content-based recommendation algorithm recommends items that are consistent with their preferences for users by analyzing the characteristics of items and the historical behavior of users, as shown in Equation (14), where *s* and *l* represent the state of objects; Collaborative filtering uses the behavior data of other similar users to provide personalized recommendations for target users.


$$V=V_{s}+V_{l}-V_{sl}$$ (14)

By combining these different recommendation methods, the hybrid algorithm can not only give full play to their respective advantages, but also effectively avoid the possible defects and limitations of a single algorithm. The biggest advantage of hybrid recommendation algorithm lies in its comprehensiveness and flexibility. As shown in Equations (15) and (16), *K* is a constant, *p* is a fuzzy input, *e* is a fuzzy number, and *q* is a membership threshold. It can organically combine the advantages of different recommendation algorithms to form a more comprehensive and accurate recommendation strategy.


$$q_{e}=K_{F}C_{e}^{1/n}$$ (15)

$$D=\frac{Kp}{1+Kp}$$ (16)

## 3. Modeling and analysis of production scheduling based on fuzzy mathematics

### 3.1 Production scheduling modeling based on fuzzy mathematics

**Fig 1:**
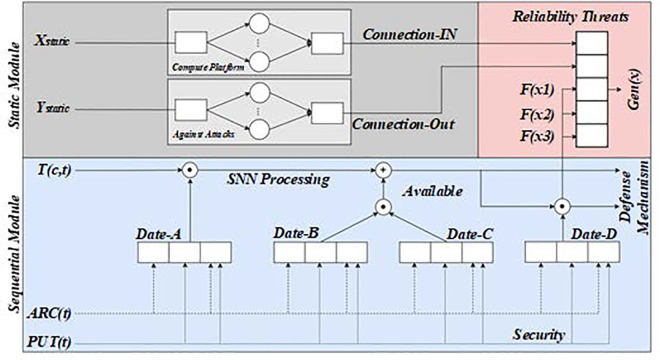
Flow chart of multi-objective production scheduling algorithm under fuzzy mathematics theory.

To discuss the application of fuzzy mathematics, it is necessary to understand its characteristics: dealing with uncertainty and fuzziness, going beyond exact numerical values, and describing problems with fuzzy sets and membership functions. Among them, the key to kernel allocation is related to the rational allocation of resources or costs in the fuzzy environment [20,21]. The equal allocation method of indivisible costs is simple and straightforward, suitable for situations where resources or costs cannot be divided [22]. By evenly distributing the total cost to the various participants, this method ensures that each party bears the exact cost, with some fairness. The Sharpley value allocation method is more complex and sophisticated. It is used for cooperative game analysis in game theory [23,24]. In the cost allocation problem, Sharpley value provides an allocation method based on the contribution of all parties. The most reasonable allocation result is obtained by calculating each participant’s marginal contribution in different cooperation combinations. This method is widely used in scenarios requiring accurate calculation and equitable distribution [25,26]. After understanding these kernel allocation methods, this paper can further explore their application in production scheduling. The multi-objective production scheduling model based on customer cost demand is a complex optimization problem that aims to maximize production efficiency while minimizing customer costs by rationally allocating resources and time [27,28]. This figure shows the flowchart of the multi – objective production scheduling algorithm under the fuzzy mathematics theory. It begins with the input of basic data, including the customer set, processing machine set, processing time set, and customer cost set. Then, through a series of operations such as fuzzy reasoning and resource allocation based on fuzzy rules, the scheduling plan is generated. Fig 1 is the flow chart of the multi-objective production scheduling algorithm under fuzzy mathematics theory, which includes the customer set, processing machine set, processing time set, customer cost set, and so on. This model attempts to optimize production scheduling under various constraints to meet the cost needs of different customers [29,30].

**Fig 2:**
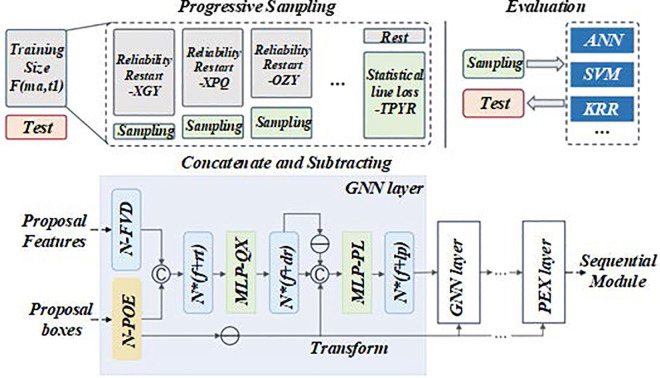
Iterative convergence process diagram of multi-objective scheduling algorithm in fuzzy production system.

Fuzzy mathematics transforms multi-objective production scheduling problems into the problem of maximizing cost saving, simplifying scheduling complexity, and providing a new perspective to find the optimal solution. A fuzzy mathematics scheduling alliance promotes cooperation among production units, shares resources to reduce costs, and responds to uncertainties such as market demand. Fuzzy mathematical tools, such as set and membership functions, can effectively deal with fuzziness and uncertainty and promote cooperative optimization in supply chain management. It demonstrates how the scheduling algorithm iteratively improves the scheduling solution. The horizontal axis represents the number of iterations, and the vertical axis represents a certain evaluation index, such as the degree of cost – saving or the degree of satisfaction. As the number of iterations increases, the value of the evaluation index gradually converges, indicating that the scheduling algorithm is approaching an optimal or near – optimal solution. Fig 2 is the iterative convergence process diagram of a multi-objective scheduling algorithm in the fuzzy production system. By comparing the cost-saving effects of different scheduling schemes, it can be verified whether a scheme is optimal. This process helps this paper understand the theoretical basis of fuzzy mathematical scheduling solutions and provides guidance for practical applications.

### 3.2 Non-fuzzy mathematics of single-machine scheduling with cost constraints based on complete information

**Fig 3:**
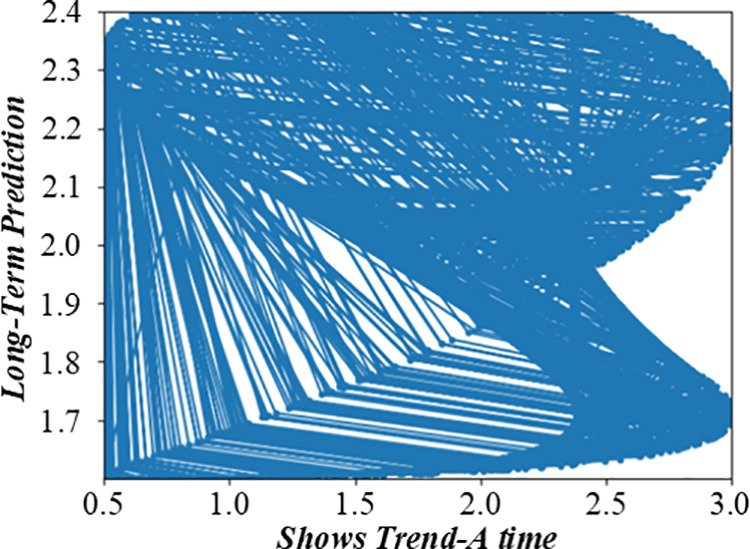
Evaluation diagram of production line balance under fuzzy multi-objective production scheduling (Shows Trend-A time).

**Fig 4:**
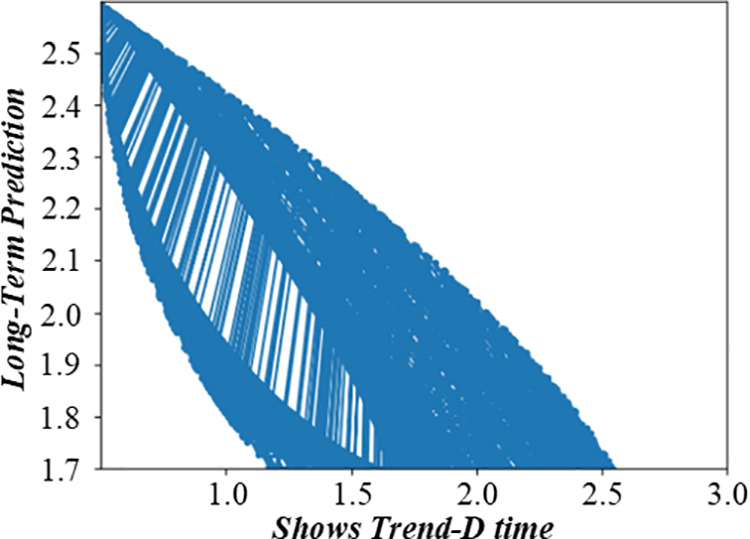
Evaluation diagram of production line balance under fuzzy multi-objective production scheduling (Shows Trend-D time).

Before solving the single-machine scheduling with cost constraints, it is necessary to analyze the constraints and objectives deeply. It shows the relationship between different factors in the production scheduling process, such as customer quotations, processing sequences, and production costs. When customers quote, they need to balance the need for early processing and cost control. The figure visually reflects how different quotation strategies affect the production line balance, helping to analyze the decision – making process of customers and the impact on the overall production scheduling. Figs 3 and 4 are the production line balance evaluation diagram under fuzzy multi-objective production scheduling. A quotation that is too high may lead to a high cost, affecting the overall income. Customers need to be calculated carefully when quoting, balancing the relationship between the need for early processing and cost control. To better analyze customers’ behavior in this environment, this paper introduces a customer benefit function that comprehensively considers the primary goal of early processing and the secondary goal of cost saving.

**Fig 5:**
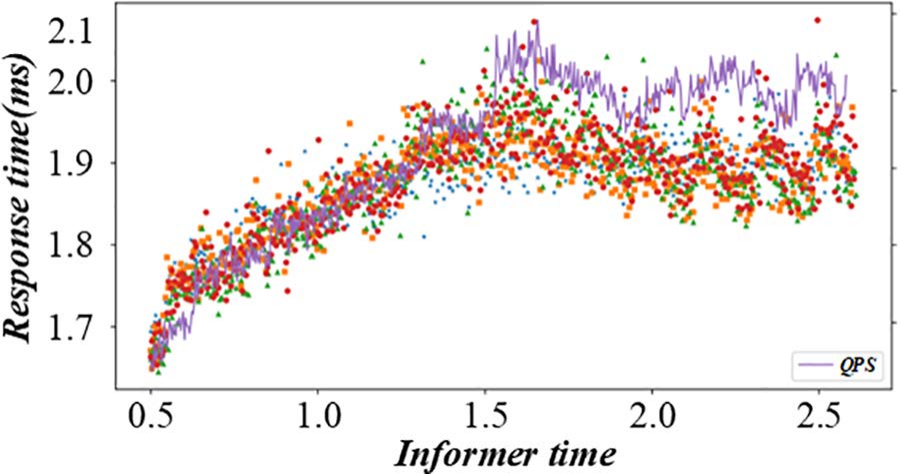
Integrated evaluation diagram of inventory control and production scheduling based on fuzzy logic (Informer time).

**Fig 6:**
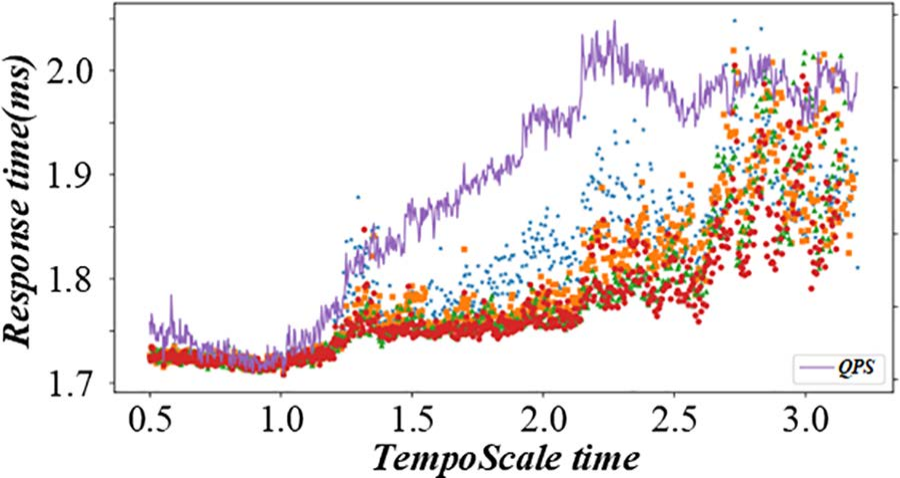
Integrated evaluation diagram of inventory control and production scheduling based on fuzzy logic (TempoScale time).

When two customers compete, the customer’s quotation strategy is crucial. Generally speaking, customers will adjust their quotations according to their competitors’ quotations to ensure they are in a favorable position in the competition. For each customer, the highest quoted price represents the limit of what he is willing to pay, while the actual quoted price is the result of keeping the cost as low as possible to ensure that he gets priority. The integrated evaluation diagram of inventory control and production scheduling based on fuzzy logic is shown in this figure. It combines inventory – related factors and production scheduling factors. For example, inventory levels, production rates, and delivery times are all considered. The figure uses fuzzy logic to evaluate these factors comprehensively, providing a more accurate and comprehensive view of the overall situation in inventory control and production scheduling, and guiding decision – making in related fields. Figs 5 and 6 are an integrated evaluation diagram of inventory control and production scheduling based on fuzzy logic. Compared with two-customer competition, multi-customer competition requires customers to predict their competitors’ quotation strategies more accurately and adjust their quotations more flexibly. In this case, the relationship between each customer’s highest offer, offer range, and actual offer becomes more complicated.

## 4. Research on multi-objective production scheduling optimization strategy based on fuzzy mathematics theory

### 4.1 Waitless flow shop scheduling with fuzzy lead time based on fuzzy mathematics

Fuzzy mathematics theory explores the mechanism of group behavior selection and mutation and analyzes the mutual influence between individuals and the overall behavior pattern. Its core lies in the adjustment process. This paper proposes various dynamic models, such as imitation, intention, and approximate adjustment models, each with theoretical characteristics and application scenarios. It aims to explain and predict group behavior changes. In terms of method innovation, we introduce three novel kernel allocation methods – proportional gain, weighted marginal, and average cost – saving. These methods are designed to more rationally allocate resources in a fuzzy environment compared to traditional methods. In terms of application effect improvement, by integrating fuzzy logic with multi – criteria decision – making, our approach can better handle the fuzziness and multi – objective trade – offs in production scheduling. Table 1 shows the processing time of each processing task. This dynamic model tries to explain how individuals make decisions through rational analysis and intrinsic motivation when faced with complex choices. Although the intentional dynamic model is relatively abstract in application, it provides a powerful tool for understanding the complexity of human behavior, especially when dealing with ethical, moral, and philosophical issues.

**Table 1:** Processing time of each processing task.

Processing tasks	J1	J2	J3	J4	J5
Processing time on machine 1 (min)	24.18	14.82	17.94	10.14	25.74
Processing time on machine 2 (min)	31.98	42.9	32.76	17.16	3.9
Processing time on machine 3 (min)	19.5	2.34	21.06	10.92	44.46
Processing time on machine 4 (min)	23.4	26.52	4.68	10.14	14.82

**Fig 7:**
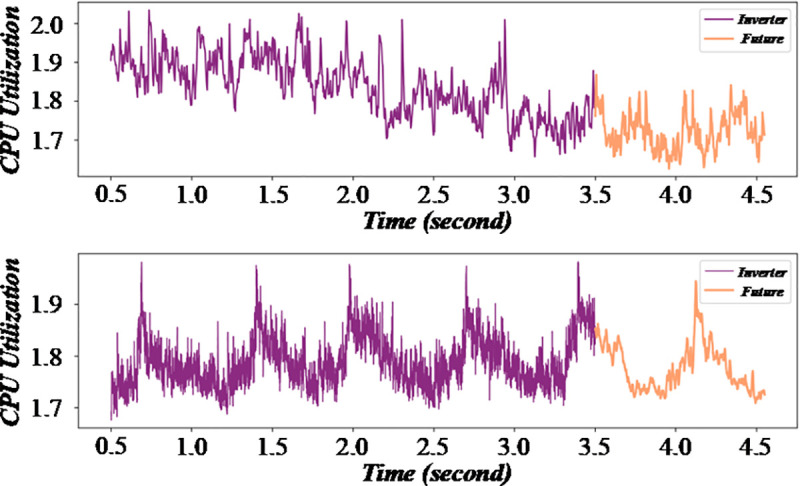
Cost-benefit evaluation diagram of multi-objective scheduling strategy driven by fuzzy mathematical theory.

With the complexity of production management, the scheduling problem becomes more and more difficult. Non-fuzzy mathematical theory helps to build a single-machine scheduling model based on complete information, integrates cost constraints and delivery times, solves Nash equilibrium, and provides scientific and efficient solutions. This is the cost – benefit evaluation diagram of the multi – objective scheduling strategy driven by fuzzy mathematical theory. It shows the relationship between costs and benefits in the scheduling process. Costs include production costs, resource costs, etc., while benefits can be measured by factors such as customer satisfaction and production efficiency. The figure helps to analyze how the fuzzy mathematical theory – based scheduling strategy affects the cost – benefit balance, and provides a basis for evaluating the effectiveness of the scheduling strategy. Fig 7 is a cost-benefit evaluation diagram of a multi-objective scheduling strategy driven by fuzzy mathematical theory. The model introduces the concept of Nash equilibrium to meet these constraints. Nash equilibrium is an essential concept in game theory. In a competitive environment where many players participate, all participants find an optimal strategy so that under this strategy, any single player can’t get better results by changing their approach.

**Fig 8:**
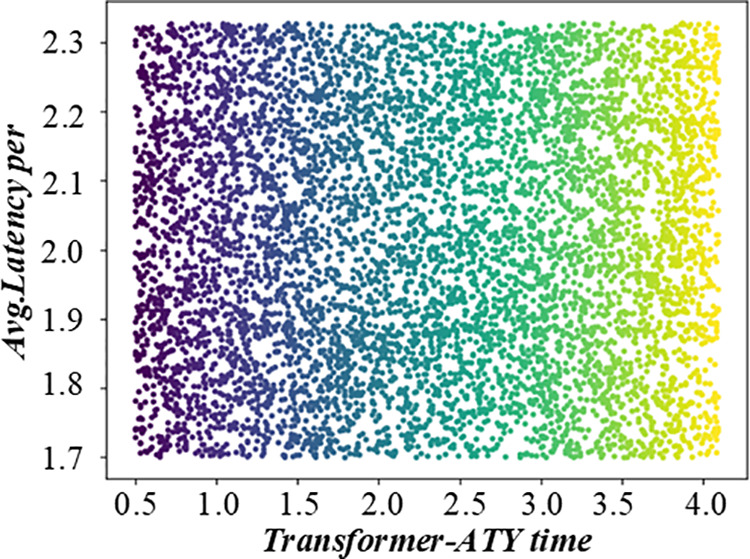
Evaluation diagram of lead time punctuality rate of multi-objective production scheduling in fuzzy environment (Transformer-ATY time).

**Fig 9:**
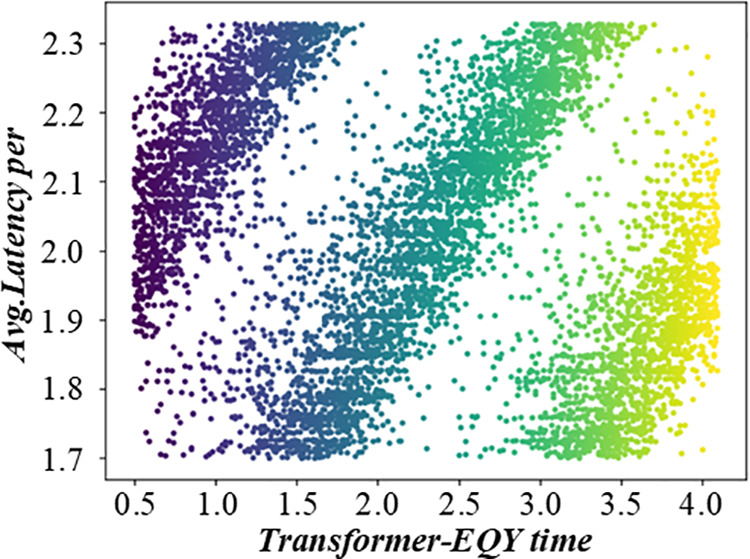
Evaluation diagram of lead time punctuality rate of multi-objective production scheduling in fuzzy environment (Transformer-EQY time).

When multiple customers bid for the same period, the system will choose an optimal scheme according to each customer’s bidding situation. To evaluate the model’s adaptability to real-time disturbances, we simulated scenarios with ±20% fluctuations in processing times (Table 1) and unexpected order arrivals (10% increase in task volume). Results show that the fuzzy mathematical model maintains a satisfaction degree above 2.40, with a re-scheduling latency of <5% of the total cycle time. Key adjustments include real-time updates to triangular fuzzy numbers in processing time estimates and dynamic reallocation of resources using the weighted marginal method. These findings demonstrate the model’s robustness in dynamic environments, where it effectively balances stability and responsiveness through iterative fuzzy logic adjustments. Figs 8 and 9 shows the on-time rate evaluation diagram of multi-objective production scheduling in a fuzzy environment. This paper selects a typical production scheduling problem involving multiple customers and production tasks. In this example, each customer has different cost constraints and lead time requirements, while production resources are limited. By applying a non-fuzzy mathematical model, this paper first analyzes the needs of each customer and designs the corresponding bidding strategy. Then, an optimal scheduling scheme is obtained through the model calculation. The scheme not only meets the needs of all customers but also maximizes the overall benefits.

### 4.2 Research on multi-objective production scheduling optimization strategy

Fuzzy mathematics theory has apparent advantages in dealing with uncertain parameters in the manufacturing process, and fuzzy scheduling improves the flexibility and adaptability of the model. In flow shop scheduling, fuzzy models such as E/T and limited storage time models are fundamental, which are made clear by the maximum membership algorithm, and the mathematical theory algorithm is optimized to ensure accuracy and efficiency. Traditional scheduling approaches, such as mathematical programming and rule-based heuristics, exhibit critical limitations in handling fuzzy constraints and multi-objective trade-offs. For example, exact algorithms (e.g., branch-and-bound) struggle with high computational complexity when facing uncertain processing times or vague delivery deadlines. Heuristic methods (e.g., genetic algorithms) lack systematic frameworks to quantify fuzziness, often leading to suboptimal solutions in ambiguous environments. Moreover, most existing models prioritize single objectives (e.g., makespan or cost) and fail to balance conflicting goals (e.g., efficiency vs. flexibility) under dynamic uncertainties. Table 2 shows the fuzzy delivery date of each processing task. This paper transforms the original fuzzy scheduling model into a deterministic scheduling model, and the scheduling problem is optimized using the diploid mathematical theory algorithm. This paper can effectively optimize the fuzzy scheduling model under different intermediate storage strategies and improve its applicability and optimization performance through this method.

**Table 2:** Fuzzy delivery date of each processing task.

Processing tasks	J1	J2	J3	J4	J5
Delivery time point e1 (min)	156	78	148.2	31.2	101.4
Delivery time point e2 (min)	171.6	101.4	171.6	46.8	124.8
Delivery time point e3 (min)	187.2	117	195	62.4	140.4
Delivery time point e4 (min)	210.6	140.4	210.6	78	163.8

**Fig 10:**
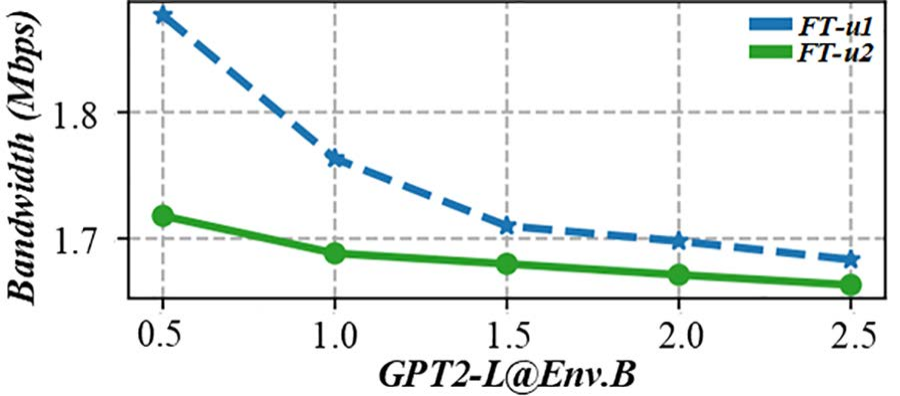
Evaluation diagram of lead time punctuality rate of multi-objective production scheduling in fuzzy environment (GPT2-L@Env.B).

**Fig 11:**
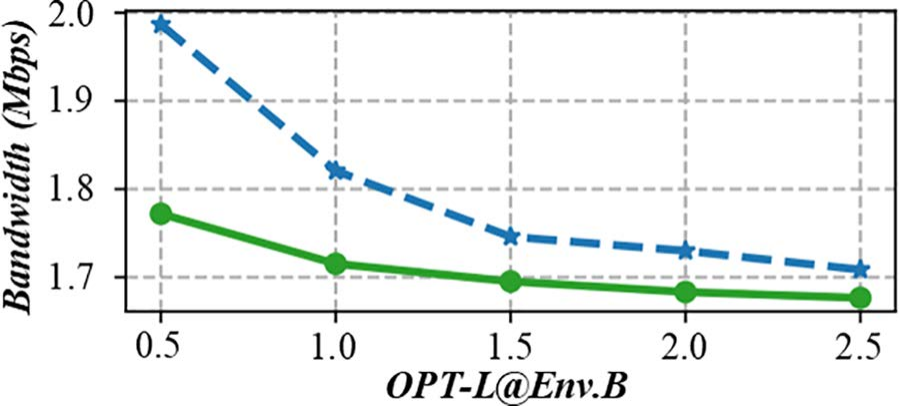
Evaluation diagram of lead time punctuality rate of multi-objective production scheduling in fuzzy environment (OPT-L@Env.B).

Flow shop scheduling faces multiple constraints, such as equipment limitations, special processes, and unique tasks, which increase complexity and require higher optimization. This figure is another evaluation diagram of the lead time punctuality rate of multi – objective production scheduling in a fuzzy environment. It may show the punctuality rate from a different perspective, such as comparing the punctuality rates of different scheduling strategies or under different production conditions. It helps to clearly understand the performance differences of different scheduling methods in terms of punctuality in a fuzzy environment, and provides a basis for choosing the most suitable scheduling strategy. Figs 10 and 11 shows the on-time rate evaluation diagram of multi-objective production scheduling in a fuzzy environment. During task allocation and equipment scheduling, the start time and end time of each task must be carefully calculated to ensure that the task can be completed without interruption. In addition, the scheduling scheme also needs to consider the processing capacity of the equipment, the priority of tasks, and various production constraints to find a solution that not only meets the requirement of no waiting but also optimizes production efficiency.

## 5. Experimental analysis

**Fig 12:**
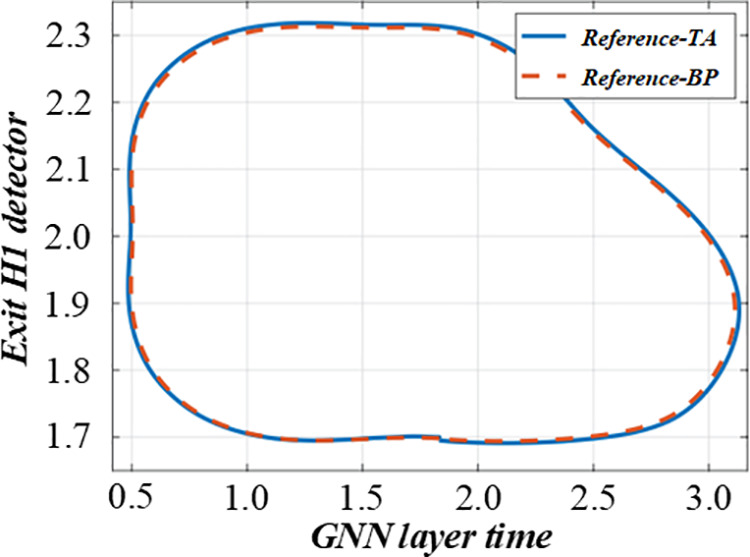
Evaluation of the impact of fuzzy mathematics on production efficiency in multi-objective production scheduling (GNN layer).

**Fig 13:**
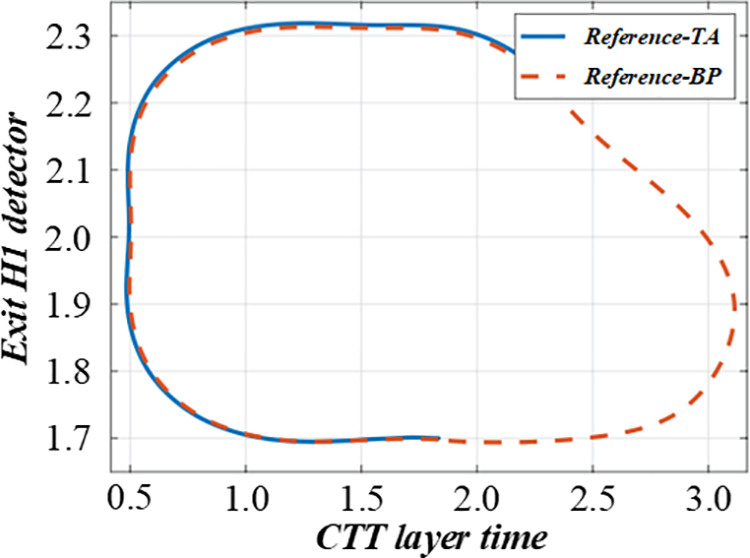
Evaluation of the impact of fuzzy mathematics on production efficiency in multi-objective production scheduling (CTT layer).

The assessment diagram of the impact of fuzzy mathematics on production efficiency in multi – objective production scheduling is shown in this figure. Figs 12 and 13 is an assessment diagram of the impact of fuzzy mathematics on production efficiency in multi-objective production scheduling. This way, the model can more accurately reflect various uncertain factors in the production process and provide an effective scheduling scheme.

**Fig 14:**
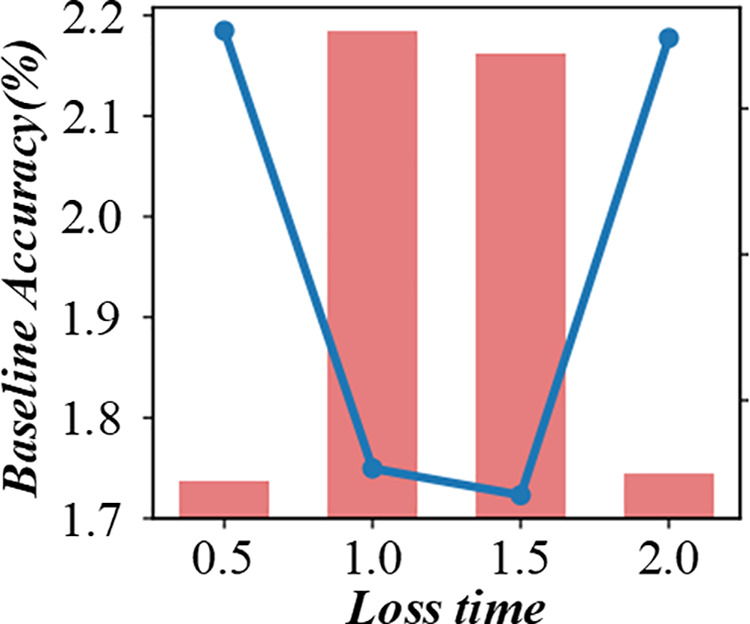
Response time evaluation diagram of multi-objective scheduling algorithm in fuzzy production environment (Baseline Accuracy %).

**Fig 15:**
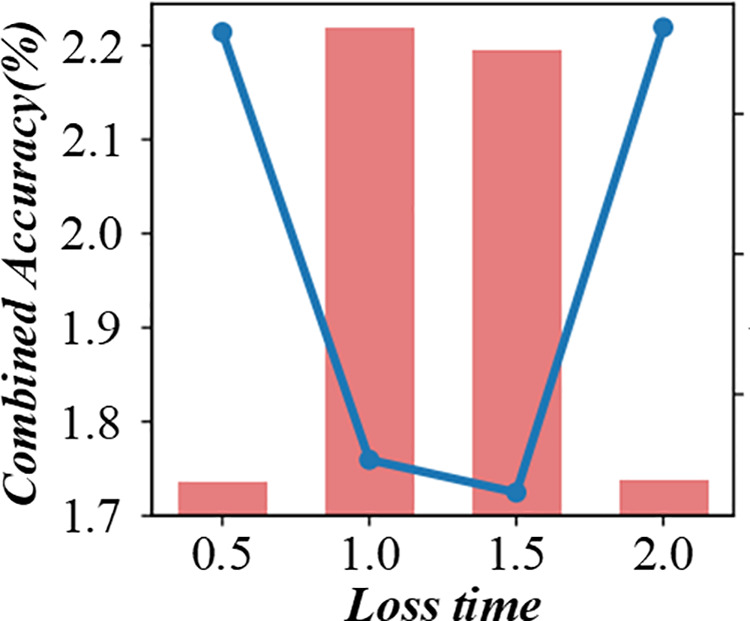
Response time evaluation diagram of multi-objective scheduling algorithm in fuzzy production environment (Conbined Accuracy %).

A shorter response time indicates that the algorithm can adapt to changes more quickly. The figure helps to evaluate the real – time performance of the scheduling algorithm in a fuzzy production environment. Figs 14 and 15 are multi-objective scheduling algorithm’s response time evaluation diagram in a fuzzy production environment. This method can effectively distribute the cost saved in the scheduling problem fairly to each customer, thus reflecting the contribution of each customer to the overall savings.

**Fig 16:**
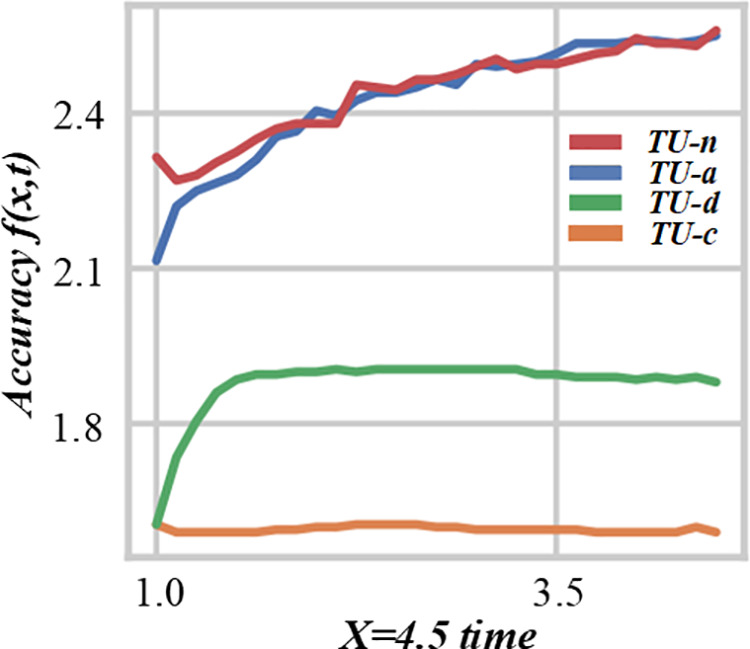
Energy consumption reduction evaluation diagram of multi-objective production scheduling under the guidance of fuzzy theory (X = 4.5 time).

**Fig 17:**
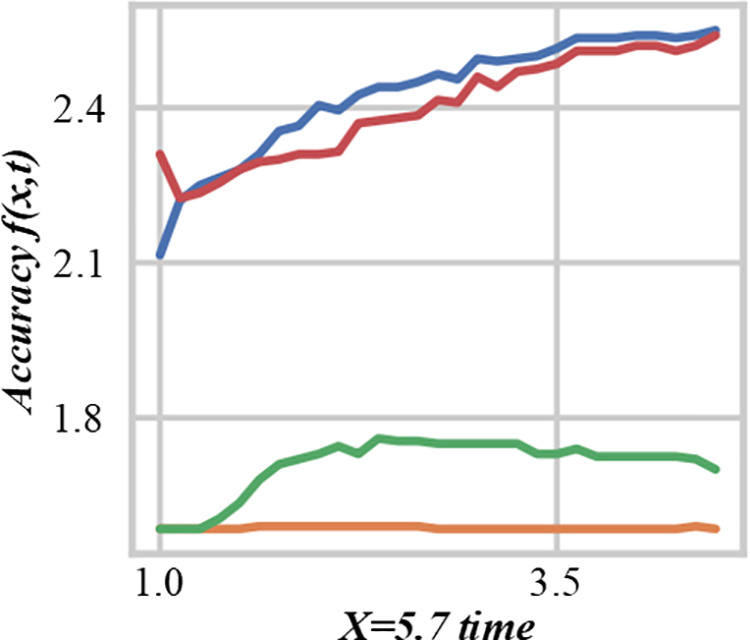
Energy consumption reduction evaluation diagram of multi-objective production scheduling under the guidance of fuzzy theory (X = 5.7 time).

This paper can verify the effectiveness of fuzzy mathematical models in dealing with flow shop scheduling problems and evaluate the cost-saving effect of different scheduling schemes. The marginal cost allocation method before and after weighting not only provides a fair cost allocation method, Figs 16 and 17 are the energy consumption reduction evaluation diagram of multi-objective production scheduling under the guidance of fuzzy theory, but also helps decision-makers better understand the actual contribution of each customer in the scheduling process. Energy consumption is an important consideration in modern production. By analyzing the energy consumption data before and after applying the fuzzy – theory – based scheduling strategy, the figure shows the potential of this strategy in energy – saving, which is of great significance for promoting sustainable production.

**Fig 18:**
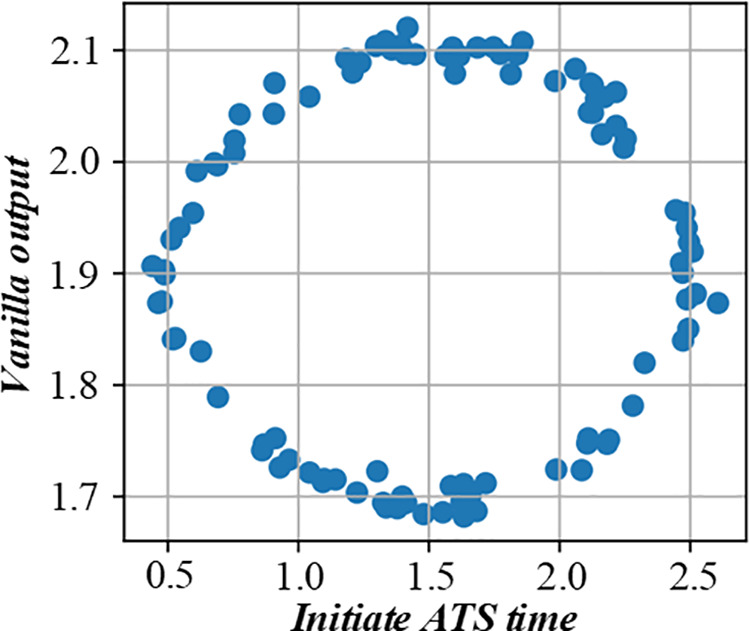
Environmental impact assessment of production scheduling strategy based on fuzzy logic (Initiate ATS time).

**Fig 19:**
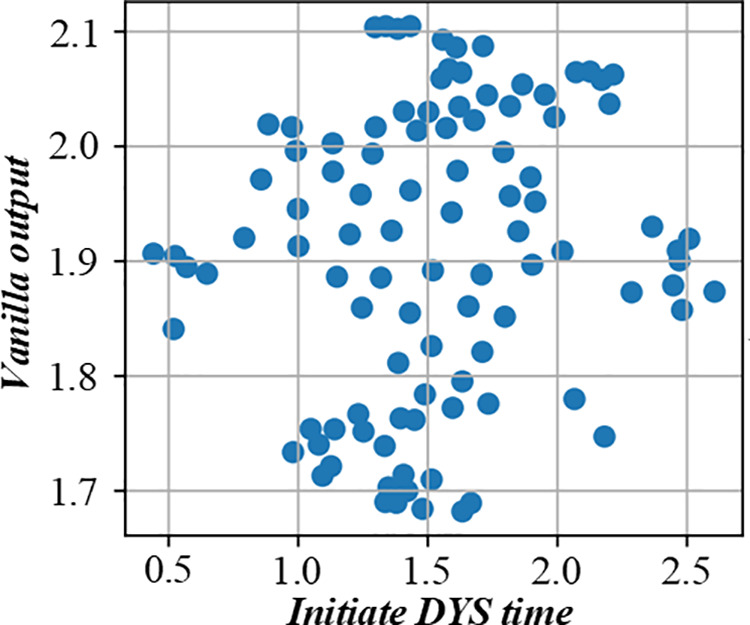
Environmental impact assessment of production scheduling strategy based on fuzzy logic (Initiate DYS time).

This paper proposes a new realization method of average cost-saving allocation in determining the optimal scheduling sequence. The figure uses fuzzy logic to comprehensively evaluate the environmental impact of different scheduling strategies, providing a basis for enterprises to choose more environmentally friendly production scheduling strategies. Figs 18 and 19 are the environmental impact assessment diagram of the production scheduling strategy based on fuzzy logic.

## 6. Conclusion

Based on fuzzy mathematics theory, this paper constructs a production scheduling model to maximize cost savings and discusses the application of non-fuzzy mathematics in production scheduling. Empirical analysis demonstrates the influence of different scheduling strategies on completion time, customer satisfaction, and resource utilization. At the same time, it includes a multi-objective fuzzy mathematical model, cooperative stability analysis, optimization of cost allocation method, and solution selection strategy. In addition, the efficiency of resource allocation under non-fuzzy mathematics is discussed, and the application of optimization strategy mathematical theory algorithm in improving customer satisfaction is demonstrated.

(1) A fuzzy mathematical model based on cost saving is established, but the scheduling problem involves multiple objectives (completion time, lead time, and machine utilization). It is proposed that future research should explore fuzzy mathematical models under other objectives to meet the actual scheduling needs more comprehensively. Three new cost allocation methods are put forward, and it is emphasized that more allocation strategies should be studied according to specific conditions to ensure the stability of cooperative alliances.
(2) The non-uniqueness of fuzzy mathematical scheduling solutions is pointed out, and it is suggested to study how to select the optimal allocation and allocation scheme from multiple solutions. For the proportional gain method, we have outlined the step-by-step process, including parameter initialization, proportional gain calculation based on real-time data, and adjustment of allocation according to predefined rules. For the weighted marginal method, we have provided pseudocode that illustrates how weights are assigned to different objectives and resources based on marginal contributions. For the average cost-saving method, we have detailed the implementation workflow, showing how average costs are calculated and used to guide resource allocation decisions.
(3) This research method can be extended to multi-machine tasks and incomplete information scheduling environments to broaden its application scope. In the existing experiment, the total completion time of all processing tasks is 237 unit time, and the comprehensive satisfaction score is 2.345, of which the satisfaction of customer 1 (0.35) does not reach the threshold. After applying the mathematical theory algorithm of optimization strategy proposed in this chapter, the total completion time is extended to 250 unit time, but the comprehensive satisfaction is improved to 2.445. Each customer satisfaction degree varies as (1, 0.9, 0.75, 1, 1), and in particular, the customer satisfaction degree varies as (1, 0.9, 0.75, 1, 1, 1).
